# Cultivating COVID-19 Vaccine Confidence in Pharmacy Professionals

**DOI:** 10.3390/pharmacy11020050

**Published:** 2023-03-07

**Authors:** Osama Aqel, Banin Alqadheeb, Mariana Felix, Collin Amundson, Jennifer M. Bingham, Katie Meyer, Terri Warholak, David R. Axon

**Affiliations:** 1 Department of Pharmacy Practice and Science, R. Ken Coit College of Pharmacy, The University of Arizona 1295 N Martin Ave, Tucson, AZ 85721, USA; 2 American Pharmacist’s Association, 2215 Constitution Avenue NW, Washington, DC 20037, USA; 3 St. Louis College of Pharmacy, University of Health Sciences & Pharmacy in St. Louis, 1 Pharmacy Place, St. Louis, MO 63110, USA

**Keywords:** vaccine confidence, pharmacists, continuing education, COVID-19

## Abstract

Pharmacists promote vaccinations and challenge misconceptions about vaccine hesitancy, yet pharmacists’ knowledge of vaccine confidence has not been assessed. The objective of this study was to compare pharmacists’ knowledge of coronavirus disease 2019 (COVID-19) vaccine confidence before and after a live continuing education (CE) session. This pretest–posttest study evaluated the differences before and after a live CE session on COVID-19 vaccine confidence provided to pharmacists at a nationwide health technology company. Participants’ total pretest and posttest scores were compared using paired *t*-tests, while pretest and posttest scores for each item were compared using chi-squared tests. A Bonferroni correction was applied, resulting in an alpha level of 0.005. A total of 279 pharmacists participated in this study. After the CE session, mean knowledge scores increased (5.2 ± 1.5 to 7.4 ± 1.35, *p* < 0.0001). After the CE session, there was no significant increase in pharmacists’ knowledge about the approach that is not recommended when discussing vaccination beliefs with a patient (71.3% to 77.4%, *p* = 0.099), determinants of vaccine uptake (83.9% to 87.8%, *p* = 0.182), and social determinants of health that can influence vaccination rates (93.6% to 96.4%, *p* = 0.121). There was a significant change in pre- and posttest knowledge for the remaining seven items.

## 1. Introduction

The Centers for Disease Control and Prevention (CDC) define vaccine confidence as the conviction that vaccines are effective, safe, and part of a trustworthy medical system [1]. Building vaccine confidence in communities that have low levels of vaccine confidence and low vaccination rates is an important public health priority [2]. Low vaccine confidence leads to vaccine hesitancy, which is one of the top 10 threats to global health according to the World Health Organization (WHO) [1], and could lead to the resurgence of disease outbreaks [3]. Since the emergence of the coronavirus disease 2019 (COVID-19) pandemic, there have been numerous studies on COVID-19 vaccine hesitancy [4]. Both the CDC and WHO support COVID-19 vaccination to gain immunity and prevent the spread of disease [5]. Vaccines have been successful in preventing or reducing morbidity and mortality from serious infections that disproportionately affect children and adults in different age groups, whereby vaccines are estimated to prevent approximately six million deaths per year and save 386 million life years globally [6]. Despite the clear and documented benefits of COVID-19 vaccines, there continues to be resistance to vaccination among many individuals in the United States (US), including healthcare workers. A pilot study on COVID-19 vaccine hesitancy among healthcare workers in the US reported that according to their participants, the common reasons for healthcare workers who reported their unwillingness to receive the COVID-19 vaccine were possible side effects, lack of fear of becoming seriously ill from COVID-19, and lack of adequate testing [7]. Many factors increased people’s COVID-19 vaccine hesitancy compared to other vaccines, including vaccine-specific factors, communication and media, historical influences, religion, culture, gender, socioeconomic factors, politics, geographic barriers, experience with vaccination, risk perception, and design of the vaccination program [8]. Building trust in COVID-19 vaccines is critical, as perceptions of vaccine safety and efficacy are strongly linked with the intent to get vaccinated.

The strategies to influence people’s confidence in COVID-19 vaccines include improving healthcare professionals’ confidence in vaccines, encouraging healthcare professionals to get vaccinated, and healthcare professionals recommending vaccines to patients [2]. A coordinated, evidence-based education, communication, and behavioral intervention could improve the success of vaccination programs [9]. Pharmacists are among the most accessible and trusted healthcare providers in their communities and are able to provide health information, answer patients’ health- and medicine-related questions, address vaccine concerns, correct misinformation, recommend the vaccine, and provide vaccinations to patients [10]. Therefore, pharmacists have an important role in promoting and supporting the uptake of vaccination and challenging misconceptions about vaccines. However, with rapid medical and technological advances, the need for lifelong learning for pharmacists is important, as it has been shown to increase the knowledge of pharmacists about several topics of importance to their practice. This is expected to have positive effects on patient outcomes, and is motivating pharmacists to enroll in continuing education (CE) sessions [11,12]. A literature review, including 185 articles from many countries, was undertaken regarding the influences of healthcare professionals’ vaccine confidence and vaccination behavior on their vaccination. Recommendations to others found that the knowledge about particular vaccines, their efficacy and safety, helped to build healthcare providers’ own confidence in vaccines and their willingness to recommend vaccines to others [13].

Previous work has explored vaccine hesitancy among healthcare professionals, and assessed pharmacists’ perceptions and acceptance of COVID-19 vaccines [14,15,16]. For instance, a national cross-sectional study assessed US pharmacists’ perceptions of COVID-19 vaccines between September and November 2020, before the emergency use authorization found that 32.9% and 36.6% of pharmacists were hesitant to receive the vaccine, and 36.6% of pharmacists were hesitant to recommend it to others [14]. A small cross-sectional study of 69 rural community pharmacists in the US conducted between late December 2020 and mid-February 2021 about the COVID-19 vaccine found that 33 pharmacists (48%) “very much” wanted to get the vaccine and 16 pharmacists (24%) “moderately” wanted to get the vaccine. The remaining 12 pharmacists (18%) expressed hesitancy toward getting the vaccine [15]. A nationwide cross-sectional study in Portugal found that 7.3% of the pharmacists who participated reported hesitancy toward vaccines; the study concluded that vaccine hesitancy is affected by perceptions, knowledge, and attitudes, and it is critical to provide interventions that focus on these determinants [16]. Furthermore, several studies assessed the CE session’s role in improving pharmacists’ knowledge on different topics [17,18,19]. For instance, one study reported that the median (range) knowledge scores of community pharmacists in Lalitpur, Nepal regarding pharmacovigilance improved significantly from a pretest median of 39 (range = 44–46) to a posttest median of 44 (range = 44–44) following an educational intervention (*p* < 0.001) [17]. Another study reported that the mean knowledge scores of pharmacists in Riyadh, Saudi Arabia regarding evidence-based medicine improved significantly from a pretest mean of 37.0% to a posttest mean of 44.4% following the research educational intervention (*p* < 0.001) [18]. Furthermore, another study evaluated the impact of a three-hour continuing education course in improving the knowledge of pharmacists to provide pharmaceutical care for transgender patients, reported a significant increase in the average total execution score from 52.15% in the pretest to an average of 72.89% in the posttest following the course (*p* < 0.001) [19]. However, no studies have yet assessed pharmacists’ knowledge of COVID-19 vaccine confidence. Knowledge of vaccine confidence is important for pharmacists to be able to provide accurate and relevant vaccine information to patients.

The purpose of this study was to compare the knowledge of COVID-19 vaccine confidence before and after a CE session on this topic among pharmacists at a Nationwide Health Technology Company in the US.

## 2. Methods

### 2.1. Study Design, Setting, and Subjects

This study used a pretest–posttest study design. Pharmacists employed at a Nationwide Health Technology Company in the US, who participated in a company-sponsored CE session designed to provide information on cultivating confidence on vaccines (described below), were eligible to participate in the study.

### 2.2. CE Presentation

CE sessions are an internationally recommended lifelong learning approach for healthcare professionals to equip them with the necessary knowledge, skills, and ethical attitudes, while keeping them current and competent in their practice [20,21]. The CE presentation was developed by a resident pharmacist at the Nationwide Health Technology Company. It was developed based on relevant literature and included information about newly approved vaccines, the effects of confidence in vaccines on vaccination rates, motivational interviewing for vaccine-hesitant patients, and tools and resources for addressing social determinants of health. The goal of the presentation was to cultivate pharmacists’ knowledge of and confidence in vaccinations. A team consisting of experienced clinical and research pharmacists reviewed and approved the final presentation.

### 2.3. Pretest–Posttest Questionnaire

The pretest–posttest questionnaire consisted of 10 knowledge-based items based on the content of the CE presentation. The questionnaire was developed in an iterative fashion by the research team, with regular input from the clinical pharmacists. The 10 questionnaire items included: (1) what percent of COVID-19 cases in the US are due to the Delta variant as of September 2021 (this item was included, as the Delta variant was the most prevalent variant around the time of the study); (2) the percentage of US adults (>18 years old) who are fully vaccinated (as of September 2021); (3) the approaches that should not be taken when discussing vaccination beliefs with a patient; (4) the recommended order in which a healthcare provider should have a conversation with a patient about vaccines, according to the WHO; (5) the most effective vaccine after >120 days of full vaccination (as of August 2021); (6) the health outcomes of myocarditis for adolescents (12–17-year-olds) who are vaccinated versus those who are unvaccinated (for every million second dose of vaccination over 120 days); (7) determinants of vaccination uptake; (8) social determinants of health that can influence vaccination rates; (9) the percentage of adults who stated that they will not get the vaccine according to the national immunization survey (as of September 2021); (10) assessment tools that can be used to identify and assess an individual’s social determinants of health. Each item had five response options. Participants were asked to select the one best response option provided for each item. Items were selected to represent the specific content covered in the CE presentation. The content validity of the questionnaire was reviewed and approved by a team consisting of experienced clinical and research pharmacists. The items had an internal consistency (Cronbach’s α) of 0.65, which indicated that the items would form an acceptable scale of moderate internal consistency [22].

### 2.4. Data Collection and Analysis

Pharmacists were invited to voluntarily participate in this study via email two weeks before the CE session. Pretest data were collected up to two weeks before the CE presentation, and posttest data were collected immediately after the CE presentation using the proprietary learning software provided by the health technology company. The pretest and posttest responses were matched using a unique identifier within the software. Each item was scored 1 or 0 depending on whether it was correct (1) or incorrect (0), and scores were summed for each subject. Scores therefore ranged from 0–10 for each subject. The mean pretest and posttest scores were compared using a paired *t*-test, where an a priori alpha level of 0.05 indicated significance. Pretest and posttest scores for each of the ten items were compared using chi-squared tests. A Bonferroni correction was applied to account for the multiple comparisons, resulting in an a priori alpha level of 0.005 (0.05/10 = 0.005) for the categorical analysis. All analyses were conducted using SAS on Demand for Academics (SAS Institute, Version 1.0.9, Cary, NC, USA).

## 3. Results

A total of 279 pharmacists from the health technology company participated in this study. The mean knowledge scores of cultivating confidence in vaccines significantly increased from 5.2 ± 1.5 before the CE session, to 7.4 ± 1.35 after the CE session (*p* < 0.0001). The percentage of correct answers for each item asked during the pretest and posttest is shown in Table 1.

After the CE session, there was a significant increase in pharmacists’ knowledge about the percentage of COVID-19 cases due to the Delta variant, the percentage of US adults (≥18 years old) who are fully vaccinated (as of September 2021), the recommended order in which a healthcare provider should have a conversation with a patient about vaccines according to the WHO, the vaccine that is most effective 120 days after full vaccination, the health outcomes of myocarditis for adolescents who are vaccinated versus those who are unvaccinated, the percentage of US adults that have stated they will not get the vaccine according to the National Immunization Survey (as of September 2021), and the assessment tool that can be used to identify and assess an individual’s social determinants of health (*p* < 0.005). However, there was no change in the proportion of correct answers pre- and posttest for the remaining items regarding the options that describe the approach not recommended when discussing vaccination beliefs with a patient (71.3% to 77.4%, *p* = 0.0099), determinants of vaccine uptake (83.9% to 87.8%, *p* = 0.0182), and the social determinants of health that can influence vaccination rates (93.6% to 96.4%, *p* = 0.0121).

## 4. Discussion

The main finding from this study was that pharmacists’ knowledge improved significantly following the CE session, which demonstrates the value of providing CE sessions to pharmacists to help improve their knowledge of COVID-19 vaccine confidence in response to the public health emergency. Although there are no other studies that report on a CE session to assess pharmacists’ knowledge of COVID-19 vaccine confidence that we can compare our findings to, this study does provide additional evidence of the value of CE sessions for pharmacists generally.

In the pretest, there were three items for which the proportion of participants who correctly answered the item exceeded 70%, and no significant improvement was seen for the posttest (the approach that is not recommended when discussing vaccination beliefs with a patient, the determinants of vaccination uptake, and the social determinants of health that can influence vaccine rates). This indicated that pharmacists already had knowledge of these aspects of vaccine confidence. A cross-sectional study undertaken in Pakistan to identify the status of knowledge, attitude, and practice regarding COVID-19 among healthcare workers reported that the majority of healthcare workers have good knowledge (93.2%, *N* = 386), a positive attitude (mean 8.43), and good practice (88.7%, *N* = 367) in regards to COVID-19 [23]. This is not surprising, as pharmacists are actively involved in seeking information due to their active roles in improving treatment outcomes of patients with COVID-19. There were two items where, although most participants (>50%) correctly answered the item in the pretest, there was a significant increase in the proportion answering the question correctly in the posttest (the recommended order in which a healthcare provider should have a conversation with a patient about vaccines according to the WHO, and the assessment tools used to identify and assess an individual’s social determinants of health). These items provide some evidence for the benefit of the CE session to help improve pharmacists’ knowledge of COVID-19 vaccine confidence. Previous literature supported the role of CE sessions in increasing pharmacists’ knowledge on various topics [17,18,19]. However, it is important to note that conducting the pretest may have had a positive impact on the posttest performance. In fact, the pretesting effect before being exposed to learning content has demonstrated an enhancement of content retention [24]. In addition, this study also showed that only a minority of pharmacy professionals (<50%) correctly answered five items during the pretest, with a significant improvement in the posttest. This finding indicates a need for further education to target knowledge gaps in pharmacists’ knowledge about COVID-19 vaccine confidence, especially as it relates to the latest data (the percentage of US adults who will not get the vaccine, the percentage of COVID-19 cases due to the Delta variant, the percentage of US adults who are fully vaccinated, and the vaccine that is most effective ≥120 days after full vaccination), and more nuanced clinical implications (i.e., health outcomes of myocarditis for adolescents). The finding that many pharmacists incorrectly answered items about the latest COVID-19 data during the pretest may be surprising, given that one would expect pharmacists to have current, up-to-date knowledge of the latest health issues. However, it is also possible that these pharmacists were working under the increased pressure of the pandemic and may not have had time to be up to date with the latest statistics, especially as the data were frequently changing at the time of the study. The finding that many pharmacists incorrectly answered the item about myocarditis for adolescents is perhaps not too surprising, given that this is a rare event in a rapidly evolving public health crisis. However, it emphasizes the need for further training on the less common implications of COVID-19. Previous research has also assessed pharmacists’ knowledge about vaccine confidence and COVID-19, and found gaps in knowledge that need to be addressed. For instance, a study of pharmacists’ role in COVID-19 in Jordan showed that pharmacists were knowledgeable about various aspects of COVID-19 (median knowledge score = 20, range = 13–25), but certain knowledge gaps regarding COVID-19 disease updates were identified that needed to be addressed [25]. Furthermore, a study of the knowledge, attitude, and practices regarding COVID-19 among pharmacists in Japan found that the mean knowledge score for questions related to COVID-19 was 4.17 ± 1.24 out of 10 points, which indicated that pharmacists needed to improve their knowledge on the topic [26].

Overall, these finding suggest that the CE sessions played a role in increasing pharmacists’ knowledge in COVID-19 vaccine confidence. Although pharmacy professionals were already knowledgeable on various aspects of the topic, there were still some knowledge gaps that require further education to improve their knowledge and better prepare them to counsel patients. Further work should be considered in developing various CE sessions on the topic of vaccination. A cross-sectional study investigating the knowledge, attitudes, and practices regarding vaccinations of community pharmacists in Italy reported that less than one quarter (23.9%) of their respondents correctly indicated the items addressing specific vaccinations for newborns (all 10 mandatory vaccinations), and nearly all of their respondents (>90%) recognized the need for further education in this field [27]. Furthermore, another nationwide cross-sectional study conducted a post-marketing survey aiming to describe the current status of the overall and profession-specific knowledge, attitudes and recommendations on the human papillomavirus (HPV) and HPV vaccines among healthcare providers in China. This study reported knowledge gaps among their participants on the risk factors of HPV infection, the best time to vaccinate, the prophylactic functions of the HPV vaccine, and in particular, the risk stratification on high-risk and low-risk types [28]. Despite this lack of knowledge among healthcare professionals, the rate of participation in organized CE sessions remains low, and little is known about the reasons for low participation rates in CE. A study undertaken in Australia to determine the barriers of pharmacists’ participation in continuing education found that the major barriers identified were time, accessibility, and relevance of content [29]. We should further encourage healthcare professionals to enroll in CE sessions to gain the opportunity to enhance their knowledge in the skills that can effect change in professional practice and, on occasion, the healthcare outcomes of their patients.

In light of the evidence of vaccination knowledge gaps among pharmacists, the spread of vaccine hesitancy among healthcare professionals, and low participation rates in CE sessions despite their benefits, the findings from this study will contribute to the literature in various ways. First, to our knowledge, this is the first study that assessed a CE session for improving pharmacists’ knowledge of COVID-19 vaccine confidence. In response to the COVID-19 public health crisis, the results from the current study demonstrated that by allowing pharmacists to obtain an educational intervention, such as a continuing education session on COVID-19 vaccine confidence, is important in enhancing pharmacists’ knowledge to provide appropriate counseling for their patients and supporting vaccination uptake. Second, the study findings provided additional evidence to the literature of the role of CE sessions in improving knowledge among pharmacists. This has been demonstrated by the significant increase in pharmacists’ knowledge of COVID-19 vaccine confidence after the CE session. Finally, the study identified various COVID-19 knowledge gaps among pharmacists before the CE session. This finding adds more to the literature regarding COVID-19 knowledge gaps among pharmacists. As pharmacists have an important role in promoting and supporting the uptake of vaccination, the need for lifelong learning for pharmacists on this topic and others is important.

Limitations of this study include the nature of the pre- and posttest research design. It is common that a pretest will likely get participants to be more aware of the test itself, and alert participants to the limited material required to score better on a posttest rather than acquiring adequate general knowledge for the subject of interest. In addition, the intervention was limited to one continuing education session, and the posttest was administrated immediately after the end of the session, which is primarily a measure of short-term memory retention. However, previous research has demonstrated that short-term knowledge gains are associated with long-term retention; this does not necessarily reflect the magnitude of knowledge acquired over the long term [30,31]. Demographic data of study participants was not collected, which may have added additional interpretation to our findings. Despite these limitations, this study highlights the importance of CE sessions to help improve pharmacists’ knowledge of COVID-19 vaccine confidence.

Future studies may include addressing items where pharmacists’ knowledge remained low, and evaluating the effect of the CE program among pharmacists at other organizations or to other healthcare professionals. In addition, pharmacists’ knowledge should be assessed over a longer time period (e.g., six months after the CE session) to measure longer-term knowledge retention. The relationship between knowledge about vaccines and their sociodemographic variables should be considered in future research as well. Continued work to raise awareness among pharmacy professionals regarding their role in educating citizens about health aspects, such as the importance of vaccination, is recommended. In addition, further assessment is needed regarding pharmacist’s skills in communication and identification of false beliefs among the population.

## 5. Conclusions

This pretest–posttest study showed that pharmacists’ mean knowledge scores of COVID-19 vaccine confidence significantly improved after attending a CE session on the topic. Further work is required to address knowledge gaps that remained after the CE session, and to expand this CE opportunity to other healthcare professionals in different settings.

## Figures and Tables

**Table 1:** Frequency and percent of a sample of pharmacists’ knowledge before and after a continuing education session about COVID-19 vaccine hesitancy (*N* = 42).

Question	Correct Answers Pretest *N* (%)	Correct Answers Posttest *N* (%)	*p*-Value
What percentage of COVID-19 cases in the US are due to the Delta variant (as of September 2021)?	37 (13.6)	184 (66.0)	**<0.0001**
What percentage of US adults (≥18 years old) are fully vaccinated (as of September 2021)?	65 (23.3)	166 (60.0)	**<0.0001**
Which approach below is NOT recommended when discussing vaccination beliefs with a patient?	199 (71.3)	216 (77.4)	0.099
Which ONE of the following options represents the recommended order in which a healthcare provider should have a conversation with a patient about vaccines, according to the World Health Organization?	158 (56.6)	238 (85.3)	**<0.0001**
Which vaccine is most effective ≥120 days after full vaccination (as of August 2021)?	132 (47.3)	222 (79.6)	**<0.0001**
Which ONE of the following options describes health outcomes of myocarditis for adolescents (12–17-year-olds) who are vaccinated versus those who are unvaccinated (for every million second dose of vaccination over 120 days)?	106 (38.0)	154 (55.2)	**0.00046**
Which ONE of the following options determines vaccination uptake?	234 (83.9)	245 (87.8)	0.182
Which ONE of the following options indicates the social determinants of health that can influence vaccine rates?	261 (93.6)	269 (96.4)	0.121
What percent of US adults have stated they will not get the vaccine according to the National Immunization Survey (**as of September 2021**)?	26 (9.3)	118 (42.3)	**<0.0001**
Which assessment tool can be used to identify and assess an individual’s social determinants of health?	221 (79.2)	251 (90.0)	**0.0004**

Differences between pretest and posttest results were compared using a chi-squared test. A Bonferroni correction was applied for the multiple comparisons, resulting in an a priori alpha level of 0.005. Bold indicates a significant difference between pretest and posttest results.

## Data Availability

The data presented in this study are available on request from the corresponding author.
